# Pediatric reference intervals for thyroid hormone levels from birth to adulthood: a retrospective study

**DOI:** 10.1186/1472-6823-8-15

**Published:** 2008-11-27

**Authors:** Klaus Kapelari, Christine Kirchlechner, Wolfgang Högler, Katharina Schweitzer, Irene Virgolini, Roy Moncayo

**Affiliations:** 1 Department of Pediatric and Adolescent Medicine, Medical University of Innsbruck, Austria; 2 Department of Nuclear Medicine, Medical University of Innsbruck, Austria

## Abstract

**Background:**

Age- and sex-specific reference intervals are an important prerequisite for interpreting thyroid hormone measurements in children. However, only few studies have reported age- and sex-specific pediatric reference values for TSH_basal _(TSH), free T3 (fT3), and free T4 (fT4) so far. Reference intervals are known to be method- and population-dependent. The aim of our study was to establish reference intervals for serum TSH, fT3, and fT4 from birth to 18 years and to assess sex differences.

**Methods:**

2,194 thyroid hormone tests obtained from a hospital-based pediatric population were included into our retrospective analysis. Individuals with diagnoses or medications likely to affect thyroid function were primarily excluded, as well as the diagnostic groups, if different from the purely healthy subgroup (n = 414). Age groups were ranging from 1 day to 1 month, 1 – 12 months, and 1 – 5, 6 – 10, 11 – 14, and 15 – 18 years, respectively. Levels of fT3, fT4 and TSH were measured on Advia^® ^Centaur™ automated immunoassay system.

**Results:**

The final sample size for reference data creation was 1,209 for TSH, 1,395 for fT3, and 1,229 for fT4. Median and 2.5/10/25/75/90/97.5 percentiles were calculated for each age group. Males had greater mean fT3 concentrations than females (p < 0.001). No sex-differences were found for TSH and fT4 between age-matched serum samples. Median concentrations of fT3, fT4 and TSH were greatest during the first month of life, followed by a continuous decline with age.

**Conclusion:**

Our results corroborate those of previous studies showing that thyroid hormone levels change markedly during childhood, and that adult reference intervals are not universally applicable to children. Moreover, differences of our reference intervals compared to previous studies were observed, likely caused by different antibody characteristics of various analytical methods, different populations or undefined geographic covariates, e.g. iodine and selenium status.

## Background

Hypothyroidism in children is associated with impaired physical and/or cognitive development. Therefore, neonatal screening for thyrotropin (TSH) values has been established in the majority of developed countries to timely diagnose and treat congenital hypothyroidism, in order to prevent severe mental retardation. During childhood and adolescence, basal TSH levels are presumed to be the most valuable parameter for diagnosing hypothyroidism and hyperthyroidism, and for the monitoring of thyroid replacement therapy. Nevertheless, measurement of free T4 (fT4) is indispensable to confirm these diagnoses, since it directly reflects hormone production by the thyroid gland [1]. Measurement of free T3 (fT3) only indirectly reflects thyroid hormone production but may provide additional information, since most of fT3 is produced by intracellular conversion by the deiodinases.

Despite their importance for interpreting individual results in clinical practice, pediatric reference values for thyroid function are scarce and published normative data often comprise only small numbers of patients [2-7]. Moreover, reference values vary considerably as they are population- and assay- specific. Significant variations of thyroid hormone levels during growth have been observed although, in contrast to a common misconception, pubertal development does not affect the concentration of thyroid hormones in children [3]. Data are inconsistent concerning sex differences. Biological variables such as nutrition and lifestyle may also influence thyroid function.

The aims of our study were 1) to establish age-specific reference intervals for serum concentrations of TSH, fT3, and fT4 in healthy children, 2) to assess sex differences in thyroid function, and 3) to compare our results to previously published reference data.

## Methods

### Subjects

Between July 2002 and October 2003 routine results of serum TSH, fT3, and fT4 were collected from existing laboratory data of 2,194 serum samples from a hospital based population of children aged 1 day – 18 years. The in- and out clinic patients had been admitted to the pediatric department of the Medical University Innsbruck for a variety of reasons. Subjects were sub-grouped according to age, ranging from 1 day to 1 month, 1 – 12 months, and 1 – 5, 6 – 10, 11 – 14, and 15 – 18 years, respectively. Classification of age groups was primarily based on those of previously published studies to facilitate reliable comparison of the results, and also specifically according to the age-related course of the obtained values. Two or more samples were taken from 410 patients, 1,085 patients had only one serum sample taken. All procedures were done in accordance with the Declaration of Helsinki.

Data were collected routinely within the setting of clinical practice according to standard procedures. The parents of the children gave their informed consent. No further measures were taken beyond clinical practice.

Classification of the patients in diagnostic categories was done using the International Classification of Diseases (ICD-10) codes. Children with conditions or concomitant medications likely to affect thyroid function were excluded from the reference group [8]. Also patients with eating disorders, with pituitary disease or chromosomal anomalies were not included in the analysis. Patients presenting a deviation in height or weight were identified at the clinical examination and classified as normal variants of growth (ICD E34.3 and E34.4) including familial or constitutional factors. All diagnostic groups have been included in a separate Excel file (additional material). Each diagnostic group was statistically evaluated in comparison to Z00.0 (n = 414; general examination and investigation of persons without complaint and reported diagnosis) using the one way ANOVA post-hoc analyses (data not shown). Serum samples from patients having the following ICD-10 diagnostic codes indicating thyroid dysfunction were excluded a-priori (n = 665): Congenital hypothyroidism with and without diffuse goiter (E03.0, E03.1), other non toxic goiter (E04.0 – E04.9), dyshormonogenetic goiter (E07.1), thyrotoxicosis (E05.0 – E05.9), autoimmune thyroiditis and unspecified thyroiditis (E06.3, E06.9), any neoplasm of the thyroid gland (C73 and D44.0), post procedural hypothyroidism (E89.0), and other specified or unspecified hypothyroidism (E03.8, E03.9).

### Methods

Analyses of TSH, fT3, and fT4 from undiluted serum samples of children and adolescents were performed on an automated immunoassay system (Advia^® ^Centaur™, Bayer Health Care Diagnostika, Vienna, Austria) using a direct chemiluminiscence detection system according to the manufacturer's instructions [9,10]. TSH was analyzed in a two-site solid-phase format, whereas analysis of fT3 and fT4 was performed in a competitive assay. The intraassay coefficients of variation for TSH, fT3, and for fT4 were < 8.7%, < 3.5%, and < 8.1% respectively. The reference values on adult patients using this immunoassay system have recently been published in BMC Endocrine Disorders and Thyroid [11,12].

### Statistical analysis

In addition to the selection criteria for the total reference group detailed above, serum values of TSH, fT3, and fT4 in each diagnostic group were also compared to the sub-group of primarily healthy children and adolescents (n = 414) using ANOVA and post-hoc Tukey's test. Groups that were not statistically different were pooled with the healthy reference group. Values of thyroid hormones were tested for their normal distribution. Because of the strong right-tailed skewed distribution of TSH values, natural log transformation was required [13,14]. Comparison between age-groups was performed using the unifactorial ANOVA test and post-hoc Tukey's test. Further post-hoc comparisons of mean values between age groups and sexes were assessed using the Student's t-test. Correlation analysis was performed using the Pearson method.

Two-tailed values of p < 0.05 were considered statistically significant. Medians and percentiles (2.5^th ^to 97.5^th^) for each variable were determined and taken as the reference interval. Statistical Package for Social Sciences (SPSS Inc., Chicago, IL, USA, version 12.0) was used for statistical analysis and creation of percentiles.

## Results

### TSH

The final sample size for the evaluation of TSH was 1,209. No difference was found between serum TSH concentrations of male (n = 636; 52.6%) and female (n = 573; 47.4%) children and adolescents (p = 0.689) of all age groups. Therefore, both sexes were combined for the calculation of percentiles. Log transformation was done before analysis because of the significant right-tailed skewed distribution of the TSH values.

**Figure 1:**
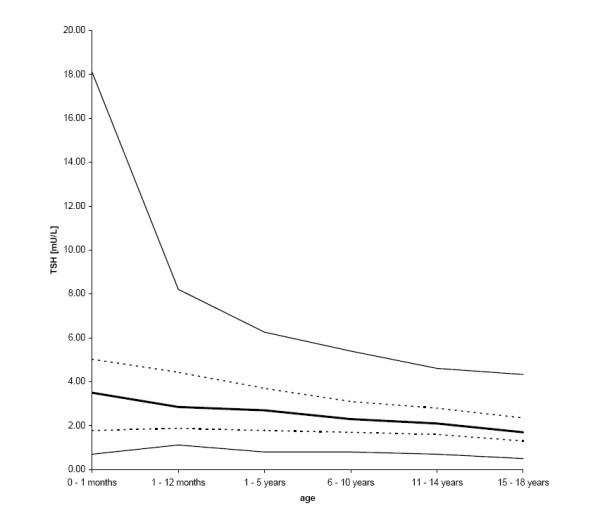
**Reference intervals for TSH of age groups listed in table 2**. The central 95% range (2.5^th^, 25^th^, 50^th^, 75^th^, and 97.5^th ^percentiles) is shown. Due to resolution reasons lines start at zero, although no samples were taken within the first hours after birth.

The 97.5^th ^percentile was significantly higher in the age group of 0 – 1 month compared to all other age groups. TSH values decreased continuously with age, particularly during the first year of life. The 2.5^th ^percentile was lower in the first month compared to the first year of life and subsequently also decreased with age (figure 1 and table 1). Variance for TSH was greatest in the first month of life and became narrower with increasing age (0–1 month: var = 15.04; 1–12 months: var = 3.55; 1–5 years: var = 2.96; 6–10 years: var = 1.46; 11–14 years: var = 1.08; 15–18 years: var = 0.98). A longitudinal sub-group of 32 patients with repeated evaluation of thyroid function showed initially elevated TSH, but normal fT3 and fT4 levels, which decreased and normalized spontaneously within 3 to 6 months without treatment. The age of these patients corresponds to that of the whole group (1 to 18 years of age). The cause for the initial elevation of TSH was not found. The follow-up evaluations discarded the presence of subclinical hypothyroidism so that their TSH values were included. The initially elevated TSH levels were detected as outliers.

**Table 1:** Percentiles for TSH (mU/L) of children and adolescents in different age groups

**Age**	**n**	**percentiles**						
		**2.5**	10	25	**50**	75	90	**97.5**
**0 – 1 months**	22	**0.70**	1.00	1.78	**3.50**	5.03	9.34	**18.10**
**1–12 months**	42	**1.12**	1.53	1.88	**2.85**	4.43	6.81	**8.21**
**1–5 years**	218	**0.80**	1.30	1.78	**2.70**	3.70	4.80	**6.26**
**6–10 years**	315	**0.80**	1.20	1.70	**2.30**	3.10	3.80	**5.40**
**11–14 years**	355	**0.70**	1.10	1.60	**2.10**	2.80	3.60	**4.61**
**15–18 years**	233	**0.50**	0.94	1.30	**1.70**	2.35	3.30	**4.33**

### fT3

**Figure 2:**
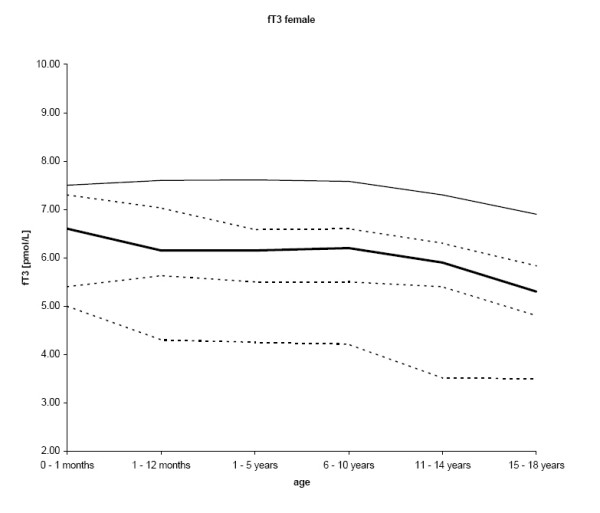
**The central 95% range for fT3 in females (2.5^th^, 25^th^, 50^th^, 75^th^, and 97.5^th ^percentiles) is shown**.

**Figure 3:**
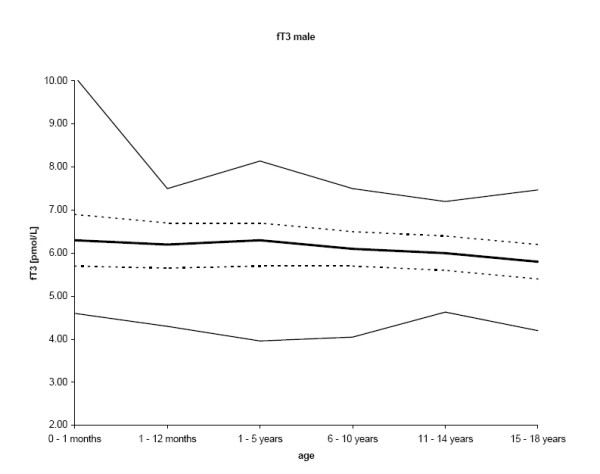
**The central 95% range for fT3 in males (2.5^th^, 25^th^, 50^th^, 75^th^, and 97.5^th ^percentiles) is shown**.

The final number of samples for fT3 was 1,395. Results for fT3 were normally distributed. As expected, the median concentrations of fT3 were greatest in the age group up to one month, thereafter showing a continuous decline with age (p < 0.001; r = -0.266 for females and r = -0.196 for males), except for males 1 – 5 years and females 6 – 10 years. Males had greater (p < 0.001) mean fT3 concentrations (n = 824; 59.1%) than females (n = 571; 40.9%). The decrease of the 2.5^th ^percentile was more distinct compared to the 97.5^th ^percentile (figures 2 and 3, table 2).

**Table 2:** Percentiles for fT3 (pmol/L) of children and adolescents in different age groups.

**Age**	**Sex**	**n**	**percentiles**						
			**2.5**	10	25	**50**	75	90	**97.5**
**0 – 1 months**	f	5	**5.00**	5.00	5.40	**6.60**	7.30	7.50	**7.50**
	m	9	**4.60**	4.60	5.70	**6.30**	6.90	10.10	**10.10**
**1–12 months**	f	14	**4.30**	4.50	5.63	**6.15**	7.03	7.50	**7.60**
	m	13	**4.30**	4.74	5.65	**6.20**	6.70	7.38	**7.50**
**1–5 years**	f	108	**4.25**	4.80	5.50	**6.15**	6.58	7.11	**7.61**
	m	111	**3.96**	5.32	5.70	**6.30**	6.70	7.48	**8.14**
**6–10 years**	f	163	**4.21**	5.10	5.50	**6.20**	6.60	7.00	**7.58**
	m	219	**4.05**	5.20	5.70	**6.10**	6.50	7.10	**7.50**
**11–14 years**	f	180	**3.51**	4.90	5.40	**5.90**	6.30	6.80	**7.30**
	m	252	**4.63**	5.20	5.60	**6.00**	6.40	6.80	**7.20**
**15–18 years**	f	98	**3.50**	4.48	4.80	**5.30**	5.83	6.50	**6.90**
	m	211	**4.20**	5.00	5.40	**5.80**	6.20	6.60	**7.47**

### fT4

**Figure 4:**
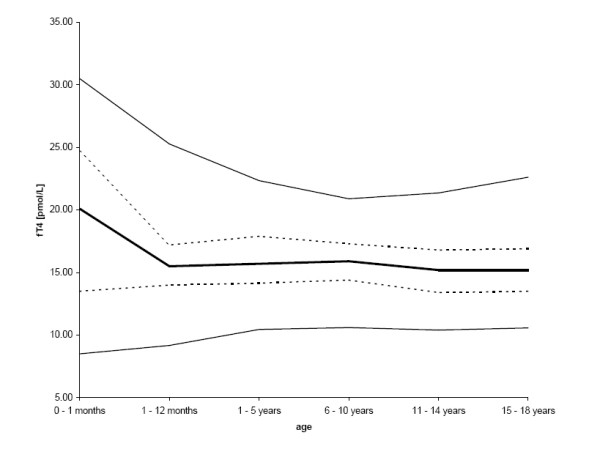
**Age-related reference values for fT4 (both sexes)**. The central 95% range (2.5^th^, 25^th^, 50^th^, 75^th^, and 97.5^th ^percentiles) is shown. Due to resolution reasons lines start at zero, although no samples were taken within the first hours after birth.

The final sample size was 1,229 for fT4 (638 males = 51.9%; 591 females = 48.1%). No sex-difference of fT4 values were found in any age group (p = 0.268) and results were normally distributed. Values for fT4 showed a significant negative correlation with age (p < 0.001; r = -0.122). Variance for fT4 was greatest in the first month of life and then became narrower with age (0–1 month: var = 45.91; 1–12 months: var = 8.60; 1–5 years: var = 7.68; 6–10 years: var = 6.13; 11–14 years: var = 8.20; 15–18 years: var = 8.08) (figure 4). The 97.5^th ^percentile showed a peak value of 30.5 pmol/L during the first month of the life and subsequently quite steady concentrations between 20 and 22.5 pmol/L. The 50^th ^percentile of fT4 was also greatest in the first month of life with a rapid decrease during the first year of life and subsequently remained constant until the age of 18 years (table 3).

**Table 3:** Percentiles for fT4 (pmol/L) of children and adolescents in different age groups.

**Age**	**n**	**percentiles**						
		**2.5**	10	25	**50**	75	90	**97.5**
**0 – 1 months**	23	**8.50**	8.98	13.50	**20.10**	24.70	28.48	**30.50**
**1–12 months**	45	**9.17**	13.10	14.00	**15.50**	17.20	19.22	**25.28**
**1–5 Years**	229	**10.45**	12.80	14.15	**15.70**	17.90	19.70	**22.35**
**6–10 Years**	327	**10.60**	12.80	14.40	**15.90**	17.30	18.90	**20.90**
**11–14 Years**	364	**10.40**	12.15	13.40	**15.20**	16,80	19.05	**21.36**
**15–18 Years**	233	**10.57**	11.74	13.50	**15.20**	16.90	18.80	**22.62**

## Discussion

This study provides new pediatric reference intervals for TSH, fT3, and fT4 for healthy Austrian children and adolescents using the Advia^® ^Centaur™ assay. Adult reference values for thyroid function are well established, although the upper limit of normal TSH values is also currently under discussion [13,15-19]. Recently adult reference values for thyroid function parameters using the Advia^® ^Centaur™ assay have been defined by our study group [11,12]. In children and adolescents, reliable age- and sex-specific reference intervals are an indispensable prerequisite to interpret individual thyroid function correctly. However, thyroid hormone values are population and method-dependent [20,21]. Thus, a series of pediatric thyroid reference data have been published using different analytical methods and antibody characteristics with variable subject numbers [2-7]. To our knowledge, only one, smaller study in children and adolescents used the Advia^® ^Centaur™ assay, which is widely used in clinical laboratories [4].

We are well aware of methodological advances in the determination of free thyroid hormones based on the technique of isotope dilution tandem mass spectrometry [22-29] which correlates better with equilibrium dialysis procedures [30]. In spite of this advantage we still advocate the used of an immunoassay procedure as we have done in this study. In previous publications we have evaluated extensively the clinical applicability of such a system for the determination of reference values for adults [11]. On practical terms, the immunoassay procedure with the Advia Centaur^® ^[5] will deliver results for fT3, fT4, TSH, thyroglobulin antibodies, and thyroid peroxidase antibodies within 20 minutes that meets requirements for clinical use [9,31]. These practical conveniences outweighs the need for a high capital expenditure [32].

One limitation of our study is that it comprises a hospital-based population. Ideally, reference intervals should be determined using blood samples obtained from a large cohort of healthy subjects. However, due to ethical and practical considerations in children, reference interval determination often relies on large hospital databases, which, by applying appropriate selection criteria, ensure sufficient number of samples [33,34]. The reference groups in this and other similar studies are not strikingly population based and results might slightly diverge. We addressed this concern by excluding all subjects with diagnoses and concomitant medications that might affect thyroid function. By this the subjects fulfilled the criteria for a healthy population as is foreseen in the category ICD10 Z00.

Median concentrations of all thyroid parameters measured were highest in the first month of life and subsequently decreased with age, apart from fT3 values of males aged 1 – 5 years and females aged 6 – 10 years. Overall, our reference data concur with those of previous studies in pediatric cohorts using various analytical methods. Slight differences of our reference intervals compared to previous studies (tables 4, 5, 6) might be explained by different analytical assays, differences in ethnicity, population or geographic derived covariates such as lifestyle, salt iodination, and nutrition. The last evaluation of iodine status done by our study group with adult patients in 2000 showed a mean value of urinary excretion of 120 μg iodine/gr creatinine (R. Moncayo, unpublished data analyzed 2002).

**Table 4:** Comparison of our TSH reference intervals to previously published reference intervals (2.5, 50 and 97.5%) of different age groups.

		**Soldin et al., 1995 Abbott IMx^®^**	**Zurakowski et al., 1999 DELFIA^®^**	**Elmlinger et al., 2001 Immulite^®^**	**Djemli et al., 2004 Access 2^®^**	**Hübner et al., 2002 Advia Centaur^®^**	**Kapelari et al., Advia Centaur^®^**
	**Age**						

**TSH**		**[mU/L]**	**[mU/L]**	**[mU/L]**	**[mU/L]**	**[mU/L]**	**[mU/L]**

		**n = 533**	**n = 5,558**	**n = 762**	**n = 706**	**n = 460**	**n = 1,209**

	**1 d – 7 d**	F: 0.72 – 13.1	n.d.	1.79 – 4.63 – 9.69	F: 1.5 – 3.3 – 6.5	0.13 – 9.23 ^a^	0.75 – 3.50 – 16.89

	**8 d–15 d**	M: 0.52 – 16.0	n.d.	1.80 – 3.71 – 7.97	M: 0.7 – 2.4 – 9.8	0.16 – 8.48 ^b^	

	**15 d – 1 m**		n.d.	n.d.			

	**1 – 12 m**		0.8 – 2.2 – 6.3		F: 1.0 – 2.4 – 5.7	0.3 – 1.73 – 5.88 ^c^	1.30 – 2.85 – 7.09

	**1 – 2 y**			0.63 – 2.04 – 4.12	M: 0.7 – 2.1 – 5.9		

	**2 – 3 y**	F: 0.46 – 8.1	F: 0.7 – 2.0 – 5.9		n.d.	0.42 – 1.98 – 4.79	1.00 – 2.70 – 5.42

	**3 – 4 y**	M: 0.55 – 7.1	M: 0.7 – 2.1 – 6.0		n.d.		

	**4 – 5 y**			0.53 – 1.60 – 2.94	n.d.		

	**5 – 6 y**	F: 0.36 – 5.8			n.d.		

	**7 – 8**	M: 0.37 – 6.0	F: 0.6 – 1.8 – 5.1	0.80 – 1.86 – 3.48	n.d.	0.48 – 1.87 – 4.67	0.90 – 2.30 – 4.40

	**9 – 10**		M: 0.7 – 1.9 – 5.4	0.85 – 2.00 – 3.50	F: 0.9 – 2.0 – 4.0		

					M: 1.0 – 1.9 – 3.7		

	**11**			0.85 – 1.90 – 3.33			

	**12**		F: 0.5 – 1.5 – 4.4	0.86 – 1.90 – 3.21	F: 0.7 – 1.7 – 3.4	0.53 – 1.78 – 4.58	0.90 – 2.10 – 4.10

	**13**		M: 0.6 – 1.7 – 4.9	0.80 – 1.81 – 3.08	M: 0.8 – 1.8 – 3.9		

	**14**			0.76 – 1.70 – 2.83			

	**15**			0.70 – 1.56 – 2.55			

	**16**			0.64 – 1.54 – 2.51	F: 0.6 – 1.5 – 3.7	0.56 – 2.00 – 4.53	0.70 – 1.70 – 3.93

	**17**		F: 0.5 – 1.3 – 3.9	0.62 – 1.51 – 2.42	M: 0.7 – 1.4 – 2.8		

	**18 – 19**		M: 0.5 – 1.6 – 4.4	0.52 – 1.36 – 2.36	n.d.		

^a ^1 d – 3 d

^b ^4 d – 30 d

^c ^61 d – 12 m

**Table 5:** Comparison of our fT3 reference intervals to previously published reference intervals (2.5, 50 and 97.5%) of different age groups.

		**Soldin et al., 1995 Abbott IMx^®^**	**Elmlinger et al., 2001 Immulite^®^**	**Hübner et al., 2002 Advia Centaur^®^**	**Kapelari et al., Advia Centaur^®^**
	**Age**				
**fT3**		**[pmol/L]**	**[pmol/L]**	**[pmol/L]**	**[pmol/L]**
		**n = 1,069**	**n = 762**	**n = 460**	**n = 1,295**
	**1 d – 7 d**	2.2 – 7.4	2.76 – 6.91 – 11.67	2.32 – 8.11 ^a^	F: 5.00 – 6.60 – 7.50
	**8 – 15 d**	2.2 – 8.4	2.84 – 5.53 – 11.86	2.40 – 7.94 ^b^	M: 4.60 – 6.30 – 10.10
	**15 d – 1 m**		n.d.		
	**1 – 12 m**	3.1 – 10.6		2.72 – 5.27 – 7.30 ^c^	F: 4.30 – 6.15 – 7.60
					M: 4.30 – 6.20 – 7.50
	**1 – 2 y**		3.28 – 6.08 – 11.06	3.05 – 5.63 – 6.93	F: 4.45 – 6.15 – 7.46
	**2 – 3 y**				M: 4.70 – 6.30 – 7.84
	**3 – 4 y**	3.7 – 10.3			
	**4 – 5 y**		3.43 – 6.57 – 10.97		
	**5 – 6 y**				F: 4.60 – 6.20 – 7.30
	**7 – 8**	4.4 – 9.2	3.58 – 6.71 – 10.75	3.30 – 5.58 – 6.79	M: 4.80 – 6.10 – 7.20
	**9 – 10**		3.72 – 6.44 – 10.87		
	**11**		3.76 – 6.13 – 9.98		
	**12**		3.76 – 5.88 – 8.86	3.46 – 5.54 – 6.7	F: 4.41 – 5.90 – 7.00
	**13**	4.8 – 9.1	3.84 – 5.64 – 8.03		M: 4.90 – 6.00 – 7.00
	**14**		3.81 – 5.45 – 7.28		
	**15**		3.69 – 5.16 – 6.87		
	**16**		3.61 – 4.98 – 6.88	3.57 – 5.33 – 6.65	F: 4.08 – 5.30 – 6.80
	**17**	5.4 – 8.8	3.54 – 4.76 – 6.60		M: 4.60 – 5.80 – 7.10
	**18 – 19**		3.52 – 4.65 – 6.30		

^a ^1 d – 3 d

^b ^4 d – 30 d

^c ^61 d – 12 m

**Table 6:** Comparison of our fT4 reference intervals to previously published reference intervals (2.5, 50 and 97.5%) of different age groups.

		**Soldin et al., 1995 Abbott IMx^®^**	**Zurakowski et al., 1999 DELFIA^®^**	**Elmlinger et al., 2001 Immulite^®^**	**Djemli et al., 2004 Access 2^®^**	**Hübner et al., 2002 Advia Centaur^®^**	**Kapelari et al. Advia Centaur^®^**
	**Age**						
**fT4**		**[pmol/L]**	**[pmol/L]**	**[pmol/L]**	**[pmol/L]**	**[pmol/L]**	**[pmol/L]**
		**n = 1,173**	**n = 353**	**n = 762**	**n = 710**	**n = 460**	**n = 1,221**
	**1d – 7d**	F: 11 – 25	n.d.	29.60 – 62.40 – 79.20	F: 11.0 – 13.6 – 22.3	10.8 – 26.8 ^a^	8.54 – 20.10 – 30.20
		M: 10 – 36					
	**8 – 15 d**	F: 8 – 25	n.d.	18.00 – 42.30 – 63.60	M: 9.8 – 12.2 – 23.2	10.9 – 25.5 ^b^	
	**15 d – 1 m**	M: 6 – 30	n.d.	n.d.			
	**1 – 12 m**	F: 11 – 24	9.5 – 19.5 – 39.5	11.10 – 19.70 – 27.30	F: 9.0 – 11.3 – 16.1	11.4 – 14.5 – 20.9 ^c^	11.95 – 15.50 – 22.51
		M: 10 – 26					
	**1 – 2 y**				M: 8.7 – 11.7 – 16.2		
	**2 – 3 y**	F: 13 – 22	9.0 – 18.4 – 37.2		n.d.	11.4 – 14.7 – 19.0	11.90 – 15.70 – 20.85
	**3 – 4 y**	M: 12 – 21			n.d.		
	**4 – 5 y**			12.90 – 17.30 – 23.90	n.d.		
	**5 – 6 y**				n.d.		
	**7 – 8**	F: 11 – 20	8.3 – 16.9 – 34.1	12.90 – 19.30 – 24.50	n.d.	11.0 – 14.2 – 18.8	11.54 – 15.90 – 19.96
	**9 – 10**	M: 10 – 22		10.30 – 17.00 – 23.80	F: 9.6 – 11.6 – 14.5		
					M: 9.7 – 11.7 – 14.2		
	**11**			11.80 – 16.70 – 22.65			
	**12**	F: 10 – 19		10.40 – 16.20 – 22.91	F: 8.8 – 10.7 – 13.5	10.8 – 13.6 – 18.7	11.10 – 15.20 – 20.00
	**13**	M: 12 – 20	7.6 – 15.5 – 31.5	8.50 – 16.50 – 22.52	M: 8.4 – 10.8 – 13.0		
	**14**			12.20 – 16.50 – 23.30			
	**15**			9.10 – 17.00 – 23.40		10.7 – 14.4 – 18.7	10.80 – 15.20 – 20.33
	**16**	F: 11 – 19		12.90 – 16.70 – 23.30	F: 8.7 – 10.7 – 13.6		
	**17**	M: 12 – 20	7.0 – 14.1 – 28.7	11.80 – 17.40 – 22.50	M: 9.5 – 11.8 – 15.0		
	**18 – 19**			9.30 – 14.50 – 20.50	n.d.		

^a ^1 d – 3 d

^b ^4 d – 30 d

^c ^61 d

Significant sex differences were observed only for fT3 in our study cohort. Interestingly, Hübner et al. found sex-specific effects also on fT3 residuals, but only within the age group 11 – 14 using the same Advia^® ^Centaur™ analyzer system [4]. Soldin et al [5] reported sex specific reference ranges without testing for statistical significance, and Djemli et al. [6] found significant differences between girls and boys on fT4 residuals only within the age group 15 – 17, whereas Elmlinger et al. [3] did not find any sex differences between sexes, also by adjusting for pubertal stage. The reason for these inconsistent findings remains unclear. Further studies in children and adolescents are needed to investigate, whether there is a significant influence of sex on thyroid hormone levels justifying distinct reference intervals.

Interestingly, in the longitudinal subgroup of 32 children with initially elevated TSH levels in the first serum sample, TSH decreased to normal without treatment within 3 – 6 months. Decision not to treat these children was based on the clinical status, since it did not correspond to subclinical or clinical hypothyroidism even though the initial TSH value was elevated. This phenomenon suggests the presence of transient benign conditions affecting thyroid function. We therefore recommend that patients with initially elevated TSH values should be retested in approximately 3 to 6 months interval, before considering replacement therapy [35]. In addition the levels of fT3, fT4, the presence of thyroperoxidase antibodies, and, in specific cases, the degree of stimulated TSH elevations should be taken into account before classifying a child or adolescent as hypothyroid [36,37]. Further prospective studies in children, similar to that of Huber et al. [38] in adults, are needed to study the spontaneous course of subclinical hypothyroidism in children and adolescents, and to determine risk factors for the development of overt hypothyroidism.

Apart from the age group 15 – 18 years, differences were observed to the published reference intervals for adult populations, especially for TSH. Recently, some authors argued for a reduction of the upper cut off range for TSH to 2.5 mU/L or somewhat more moderate to 3.0 mU/L in adults [15,16,39,40]. Applying this upper range to pediatric patients might lead to a considerable high number of false diagnoses of hypothyroidism and result in unnecessary lifelong replacement therapy. A similar scenario has already been described for TSH reference values for adults [41].

In conclusion, this study provides pediatric reference intervals for thyroid function tests from birth to young adulthood for a middle European population. Thyroid hormone levels markedly change during childhood without showing significant sex differences, except for fT3. Moreover, differences of our reference intervals compared to previous studies were observed, likely caused by different antibody characteristics of various analytical methods, different populations or unknown geographic covariates. Our results argue in favor that adult reference intervals are not universally applicable to children. These reference intervals may serve as a tool for the correct interpretation of individual thyroid hormone results of children and adolescents.

## Conclusion

Our results corroborate those of previous studies showing that thyroid hormone levels change markedly during childhood, and that adult reference intervals are not universally applicable to children. Moreover, differences of our reference intervals compared to previous studies were observed, likely caused by different antibody characteristics of various analytical methods, different populations or undefined geographic covariates, e.g. iodine and selenium status.

## Abbreviations

TSH: thyrotropin; fT3: free trioiodithyronine; fT4: free thyroxine.

## Competing interests

The authors declare that they have no competing interests.

## Authors' contributions

KK drafted the manuscript, designed and coordinated the study together with RM. CK collected retrospectively patient data and performed statistical analysis together with RM. RM and IV were in charge of the immunoassays. WH and KS participated in patient data collection. All authors read and approved the final manuscript.

## Pre-publication history

The pre-publication history for this paper can be accessed here:


